# Subfoveal Choroidal Thickness After Femtosecond Laser-Assisted Cataract Surgery for Age-Related Cataracts

**DOI:** 10.3389/fmed.2022.826042

**Published:** 2022-07-04

**Authors:** Wan Chen, Hui Chen, Lan Mi, Jing Li, Haotian Lin, Weirong Chen

**Affiliations:** ^1^State Key Laboratory of Ophthalmology, Zhongshan Ophthalmic Centre, Sun Yat-sen University, Guangzhou, China; ^2^Center for Precision Medicine, Sun Yat-sen University, Guangzhou, China

**Keywords:** femtosecond laser-assisted cataract surgery (FLACS), subfoveal choroidal thickness (SFCT), foveal retinal thickness (FRT), conventional phacoemulsification surgery (CPS), aqueous flare

## Abstract

**Purpose:**

To compare the effects of femtosecond laser-assisted cataract surgery (FLACS) and conventional phacoemulsification surgery (CPS) on subfoveal choroidal thickness (SFCT) in patients with age-related cataracts.

**Methods:**

In this prospective consecutive study, 26 patients (26 eyes) with age-related cataracts without previous ocular surgery or other ocular diseases who had FLACS were included as the study group. Twenty-six age-matched patients (26 eyes) who underwent CPS in the same period were also included as the control group. The SFCT and the foveal retinal thickness (FRT) were measured at baseline and at 1 day (D1), 7 days (D7), 1 month (M1), and 3 months (M3) postoperatively by spectral-domain optical coherence tomography. Aqueous flare was also measured with a laser flare meter.

**Results:**

The mean SFCTs of the FLACS group at baseline and at D1, D7, M1, and M3 were 185.2, 174.3, 184.2, 180.8, and 184.1 μm, respectively. A Bonferroni posttest showed that the choroid became thinner on postoperative D1 (*P* = 0.006). The measurements at 1 week, 1 month, and 3 months postoperatively showed no significant differences in the SFCTs compared with that at baseline (*P* = 0.66, *P* = 0.22, and *P* = 0.53, respectively). A different trend was observed in the CPS group. The choroid became thicker by the 3-month postoperative measurement, as follows: 1 day (*P* = 0.28), 1 week (*P* = 0.016), 1 month (*P* = 0.020), and 3 months (*P* < 0.001).

**Conclusion:**

The mean SFCT significantly and temporarily decreased following FLACS. In contrast, an increased SFCT was observed following CPS.

## Introduction

The choroid being the most vascular tissue in the eye provides nutrition to the outer retina. Its role in various chorioretinal diseases such as choroidal neovascularization (1), high myopia-related choroidal atrophies (2), polypoidal choroidal vasculopathy (3), and central serous chorioretinopathy (4), have been well established. Studying the choroidal structures may provide further understanding of the pathology and mechanisms of the related underlying diseases. In 2008, a new method for visualizing the choroid, that is, enhanced-depth imaging (EDI) optical coherence tomography (OCT), has been reported (5). EDI is an innovation of image acquisition and processing that has been applied in commercial spectral-domain OCT (SD-OCT) devices. Using EDI, deep choroidal images are enhanced by the acquisition of inverted images and the use of multiple B-scan averaging to improve the signal-to-noise ratio (SNR). SD-OCT with the EDI technique has become an established method that is widely used to measure choroidal thickness for clinical and research applications (4, 6).

Conventional phacoemulsification surgery (CPS) was widely used worldwide due to the mature technique and lower complication rates. However, pseudophakic cystoid macular edema (PCME) is still a primary cause of visual loss postoperatively. Although the exact pathogenesis of PCME is not clear, several previous studies have reported a significant increase in choroidal thickness after CPS (7, 8). In Fleissig et al.’s study, choroidal thickness was increased in cases of PCME and decreased after edema resolution (9). In Xu et al.’ study, lens extraction elicited an acute pro-inflammatory gene and protein response in both retinal and choroidal tissue of the eyes in rodents (10). These previous studies indicated that the choroidal layer may participate in the inflammatory reaction after cataract surgery.

Recently, femtosecond laser-assisted cataract surgery (FLACS) has become an accepted method of surgical intervention that has several advantages (11, 12). The prominent advantage of FLACS has been suggested to be the good reproducibility and precision of the anterior capsulotomy size and centration (13, 14). In addition, FLACS reduces the cumulative dissipated energy that might reduce the release of inflammatory mediators, which might result in less macular effect (15, 16). Recently, Asena et al. conducted a study to compare the short-term changes in the retina and choroidal tissue after FLACS and CPS (17). However, it is not clear whether FLACS alters retinal and choroidal thickness in long-term outcomes. In addition, the association between the response in the posterior segment and the inflammatory reaction after FLACS was largely unknown. The goal of the present study was to determine the changes in retinal and choroidal thicknesses in patients who have undergone FLACS compared with patients who have undergone CPS and to explore the potential factors associated with the retinal and choroidal thicknesses.

## Materials and Methods

All procedures in the present study adhered to the tenets of the Declaration of Helsinki and were approved by the Institutional Review Board of Zhongshan Ophthalmic Center, Sun Yat-sen University, China. Informed consent was obtained from each of the enrolled patients. Twenty-six eyes of 26 consecutive patients with cataracts who underwent FLACS included as the study group. The cataract stages of the patients included were between N2 and N4 according to the LOCS III (18). The exclusion criteria were eyes with a history of ocular surgery, trauma, and ocular diseases, including diabetic retinopathy, high myopia, age-related macular degeneration (AMD), pseudoexfoliation, uveitis, retinal vein occlusion, and other inflammatory and vascular retinal disorders. In addition, 26 eyes of 26 age-, sex- and cataract staging-matched patients with cataracts who underwent CPS were included as the control group in the same period. The exclusion criteria for this group were identical to those previously described. Patients with poorly dilated pupils and those with floppy-iris syndrome were also excluded from both groups.

### Surgical Procedures

All surgeries were performed by the same surgeon (WR.C) at the Zhongshan Ophthalmic Center, Guangzhou, China.

### Femtosecond Laser-Assisted Cataract Surgery

A femtosecond laser (Alcon-Lensx, Alcon-Lensx, Inc.) was used in this study. After pupil dilation, one drop of tropicamide (0.5%) was applied every 15 min for three applications, and topical anesthesia with proparacaine hydrochloride (0.5%) was applied. The laser was docked to the eye using a curved contact lens to applanate the cornea. The anterior capsulotomy (size 5.0 mm, 6 μJ pulse energy), lens-fragmentation (10 μJ pulse energy), primary and side-port corneal incisions (6 μJ pulse energy) were created under Optical Coherence Tomography image control. Then a standardized lens-fragmentation pattern (3 cross-sections with a chop diameter of 5.2 mm and 1 central chop cylinder with a diameter of 3.0 mm) was used. The laser was disconnected, and the remainder of the surgery was performed as phacoemulsification (Infiniti Vision System; Alcon, United States). A single-piece AcrySof SN60WF IOL was implanted in the capsular bag.

### Conventional Phacoemulsification Cataract Surgery

Phacoemulsification was performed through a temporal 3.2-mm clear corneal incision with the same machine and followed by implantation of IOLs. A single-piece AcrySof SN60WF IOL was implanted in the capsular bag.

Complications including iris trauma, posterior capsule rupture, vitreous loss, and prolapse through the wound were the exclusion criteria, although none of the enrolled patients experienced surgical complications.

### Spectral-Domain Optical Coherence Tomography

The subfoveal choroidal thickness was measured using SD-OCT (Heidelberg Engineering, Heidelberg, Germany). The EDI images were averaged based on 100 scans using an automatic averaging and eye-tracking system. The subfoveal choroidal thickness (SFCT) was defined as the vertical distance between the retinal pigment epithelium (RPE) and the choroidal–scleral interface at the fovea. The horizontal section running through the center of the fovea was selected for further analysis. The retinal thickness was determined as the vertical distance between the internal limiting membrane (ILM) and the RPE (19).

The measurements were performed by two independent examiners using calipers and the Heidelberg Eye Explorer software (version 5.4; Heidelberg Engineering Co.). The measurements from the two examiners were averaged. The SFCT and foveal retinal thickness (FRT) values were measured before surgery, and at 1 day (D1), 7 days (D7), 1 month (M1), and 3 months (M3) after cataract surgery. The measurements were performed between 9:00 and 11:00 a.m. due to the previously reported circadian variations in choroidal thickness (20, 21).

### Measurement of Aqueous Flare

Aqueous flare was measured with a laser flare meter (FC-2000, Kowa, Tokyo, Japan) as described in a previous study (22). Measurements were taken within 1 week before surgery and at D1, D7, M1, and M3 after surgery. Flare values were measured under scotopic conditions without pharmacologic pupil dilation. Two different examiners obtained five measurements from the studied eye, then the results were averaged after excluding all measurements affected by artifacts.

### Statistical Analysis

The SFCT results were the basis for the calculation of the sample size. Based on pretest results, the between-group difference in the mean SFCT in 3 months after surgery was estimated to be 15.2 μm, and the standard deviation was 16.6 μm. Using PASS (PASS 11, NCSS, Kaysville, UT, United States) to calculate the sample size, the outcome was that a total of at least 20 participants were required to be included in each group (*a* = 0.05, power = 0.8) (23). After assuming a 20% dropout rate, it was necessary to enroll at least 25 individuals per group in the study. We added the description of sample size calculation to the methods. The data were analyzed using the Statistical Package for Social Sciences (SPSS Version 19.0 for Windows). Descriptive statistics were used to summarize the patients’ demographic and clinical information. All data are expressed as mean values ± standard deviations. Changes in the SFCT and FRT values following cataract surgery were analyzed with repeated-measures analysis of variance (ANOVA) with Bonferroni posttests. The differences in the SFCT and FRT values between the two groups were analyzed with independent *t*-tests. Correlation analyses were used to evaluate the strengths of the associations between the magnitude of the change in SFCT from baseline to the peak and the potential explanatory variables. A *P*-value of less than 0.05 was considered statistically significant.

## Results

The FLACS group comprised 26 eyes from 26 patients (12 males and 14 females), with an average age of 68.2 ± 7.7 years. The CPS group comprised 26 eyes from 26 patients (11 males and 15 females), with an average age of 68.3 ± 6.5 years. The patient demographics are presented in Table 1. No statistically significant differences between the groups were noted in terms of age, sex, cataract staging, axial length (AL), or preoperative SFCT or FRT (*P* > 0.05). All patients fulfilled the evaluation visits before and after surgery.

**TABLE 1:** Demographic and clinical parameters of the FLACS and CPS groups.

Variables	FLACS	CPS	*P*
Age	68.2 ± 7.7	68.3 ± 6.5	0.80
Gender, male, n (%)	12 (46%)	11 (42%)	0.78
LOCS III NO score	3.0 ± 0.73	3.1 ± 0.85	0.65
AL	23.8 ± 1.1	23.5 ± 0.7	0.24
IOP	13.4 ± 3.4	13.9 ± 3.0	0.60
Phacoemulsification time	30.2 ± 20.2	43.4 ± 19.5	0.04*
Phacoemulsification energy	13.5 ± 6.4	27.0 ± 7.9	<0.01*
SFCT baseline	185.2 ± 75.8	177.5 ± 69.9	0.70
FRT baseline	205.0 ± 22.4	195.5 ± 26.3	0.25
**Aqueous flare**			
Preoperative	5.5 ± 1.9	5.0 ± 1.4	0.25
**Postoperative**			
1 Day	16.5 ± 3.8	21.7 ± 9.2	0.03*
7 Days	9.5 ± 1.6	12.2 ± 2.2	<0.01*
1 Month	6.6 ± 1.4	8.1 ± 1.3	<0.01*
3 Months	5.2 ± 1.6	5.1 ± 1.8	0.88

*FLACS, femtosecond laser-assisted cataract surgery; CPS, conventional phacoemulsification surgery; AL, axial length; SFCT, subfoveal choroidal thickness; FRT, foveal retinal thickness.*

*p-value < 0.05.

The phacoemulsification time and energy were significantly greater in the CPS group than in the FLACS group (*P* = 0.04 and *P* < 0.001, respectively). The difference in preoperative aqueous flare was not statistically significant between the groups. However, the postoperative aqueous flare was significantly greater in the CPS group than in the FLACS group at D1 (*P* = 0.03), D7 (*P* < 0.001), and M1 (*P* < 0.001) after surgery (Table 1). In M3, the difference between the groups was insignificant (*P* = 0.88).

**FIGURE 1:**
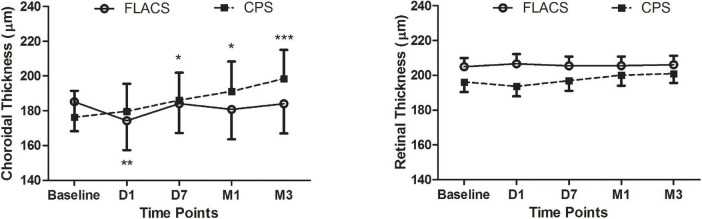
Time courses of the subfoveal choroidal (left) and retinal (right) thicknesses before and after cataract surgery. Repeated-measures ANOVA of the choroidal changes indicated significant changes in both groups (both *P* < 0.01). The SFCT in the CPS group exhibited a trend toward an increase over time (P_*D1*_ = 0.28, P_*D7*_ = 0.016, P_*M1*_ = 0.020, and P_*M3*_ < 0.001). In contrast, the SFCT of the FLACS group did not change with the exception of a slight decrease at postoperative D1 (P_*D1*_ = 0.006, P_*D7*_ = 0.66, P_*M1*_ = 0.22, and P_*M3*_ = 0.53). In contrast, repeated-measures ANOVAs of the retinal changes were not significant in either group (P_*FLACS*_ = 0.47 and P_*CPS*_ = 0.39). *p-value < 0.05, **p-value < 0.01, ***p-value < 0.001.

The Bonferroni *post hoc* tests showed that the SFCT of the FLACS group became thinner only at D1 (*P* = 0.006, Figure 1) postoperatively, then stayed no difference with the preoperative values from 1 week to 3 months. In contrast, the choroidal thickness exhibited significant variations from 1 week until 3 months (*P* = 0.001) in the CPS group. The choroid was significantly thicker at D7 (*P* = 0.016), M1 (*P* = 0.020), and M3 (*P* < 0.001) postoperatively (Figure 1).

**FIGURE 2:**
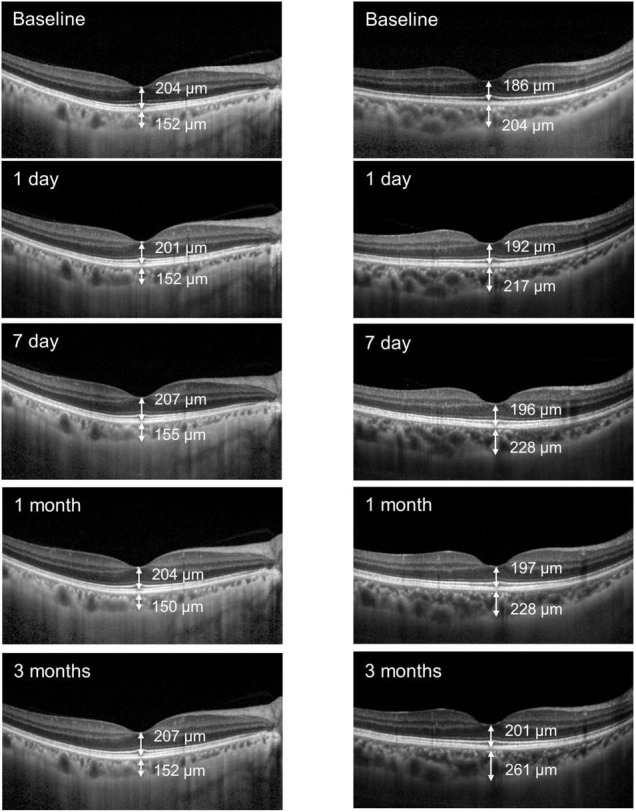
Two representative cases illustrating the choroidal thickness and retinal thickness measurements before and after FLACS (left) and CPS (right) surgery.

There were no significant differences in the mean FRT between the groups (*P* > 0.05, Figure 1). Figure 2 illustrates the SFCT and FRT measurements of two representative FLACS and CPS group cases before and after cataract surgery.

Table 2 presents the results of the correlation analyses of various factors, including age, AL, preoperative intraocular pressure (IOP), preoperative flare, phacoemulsification time, phacoemulsification energy, change in IOP, and change in flare, with the peak change in the SFCT (D1 in the FLACS group and M3 in the CPS group). All the variables were not significantly correlated with the peak changes in SFCT in the two groups.

**TABLE 2:** Correlations of various factors with the peak change in the subfoveal choroidal thickness in the FLACS and the CPS group.

	FLACS		CPS	
	*r*	*P*	*r*	*P*
Age	–0.13	0.58	–0.34	0.14
AL	0.16	0.49	–0.10	0.66
Preoperative IOP	–0.14	0.55	–0.13	0.59
Preoperative flare	–0.34	0.14	0.14	0.56
Phacoemulsification time	–0.15	0.53	0.21	0.37
Phacoemulsification energy	0.09	0.70	0.03	0.90
Change in IOP	–0.13	0.59	0.11	0.63
Change in flare	0.35	0.13	–0.17	0.46

*FLACS, femtosecond laser-assisted cataract surgery; CPS, conventional phacoemulsification surgery; AL, axial length.*

Up to the last visit, none of these cases had postoperative complications, including PCME in the two groups.

## Discussion

In our prospective study, we found that the mean SFCT, as observed on EDI-OCT, showed no difference with the baseline value at 1 week after FLACS. In contrast, we observed a tendency toward an increased SFCT in the CPS group, which is in agreement with the findings of previous studies. Recently, Asena et al. have demonstrated that the central choroidal thickness did not change postoperatively in the FLACS group. However, the mean SFCT at 1 day, 1 week, and 1 month after CPS were significantly higher than that at the baseline (17). Noda et al. found an increased SFCT for at least 6 months after cataract surgery in 29 patients with senile cataracts (7). Ohsugi et al. reported a significant increase in choroidal thickness after cataract surgery starting at 3 days postoperatively and extending up to 6 months (8).

Increased choroidal thickness is found in several diseases, including exudative age-related macular degeneration, polypoidal choroidal vasculopathy, idiopathic choroidal neovascularization, central serous chorioretinopathy, and posterior uveitis, although the reason for the increased vascular permeability of the choroid is yet to be determined. Recently, Fleissig et al. found that PCME went along with transient changes in choroidal thickness, the choroidal thickness was increased in cases of PCME and decreased after edema resolution (9). The mechanism of the change in SFCT following cataract surgery is not fully understood. One speculation is that these changes may be related to postoperative inflammation because proinflammatory prostaglandins and cytokines are thought to cause macular edema after cataract surgery, and inflammatory disorders are also known to increase the choroidal thickness (24, 25). In the present study, none of these cases had PCME up to the last visit. Whether the choroidal thickness changes after cataract surgery correlated with PCME warrants further studies with a larger sample size.

In the present study, we found that the aqueous flare was significantly minor in the FLACS group than that in the CPS group in the early postoperative stage. Further, at 3 months post-surgery, there were no differences between the two groups. Similarly, Chen et al. found that postoperative aqueous flare was significantly greater in a manual cataract surgery group than in a FLACS group (26). In that study, the authors also found discrepant expression of cytokines between the two groups based on the analyses of 45 human aqueous humor mediators, including higher levels of fibroblast growth factor and tumor necrosis factor-α and lower levels of interleukin-9 and platelet-derived growth factor-BB in the FLACS group (26). In a recent study, Liu et al. found the lower aqueous malondialdehyde but higher prostaglandin E_2_ and interleukin-1 receptor in the FLACS group compared with the conventional group (27). Also, evaluated interleukin-6 level was found in the FLACS group compared to controls (28). Nomura et al. demonstrated that higher concentrations of CXCL13 were correlated with thicker subfoveal choroidal thicknesses in 76 eyes of cataract patients (29). As documented in a previous study (11), FLACS procedures diminish the phacoemulsification time and energy used in the surgery. However, we did not establish the relationships between the phacoemulsification time and energy with the subfoveal choroidal thickness in the present study. Therefore, whether these phacoemulsification parameters play a role in the alteration of the choroidal thickness warrants further studies.

The choroidal thickness exhibited a transient decrease in the FLACS group at D1 after surgery, which may have been due to the increased IOP during the suction. Many previous studies have demonstrated that increased IOP during the suction phase of FLACS is statistically significant compared to baseline IOP, including in porcine models and patients with cataract (30, 31). It may be speculated that a decrease in ocular perfusion pressure due to increased IOP might have influenced choroidal thickness. As demonstrated in a previous study, increased IOP during suction is temporary, and IOP at 1 h after surgery is not significantly higher than the preoperative values (30, 31); this might explain the transient decrease in the SFCT in the present study.

As with most studies, our findings need to be considered in light of our study limitations. First, we measured only the SFCT 3 months postoperatively. However, we demonstrated the differences between groups, and imaging choroidal thickness maps may provide more information regarding the overall changes. Second, the sample size in the present study was small; however, we demonstrated significant variations over time in the CPS group. Further studies with larger sample sizes may confirm our findings from the FLACS group.

## Conclusion

In conclusion, this study demonstrated that the SFCT temporarily decreased in patients who underwent FLACS, whereas a significant increase was evident in the CPS group. The aqueous flare was significantly minor in the FLACS group compared with the CPS group in the early postoperative stage, and similar at 3 months postoperatively. Future investigations should consider whether FLACS is beneficial, for FLACS and CPS have similar excellent safety and refractive outcomes, while FLACS is shown less cost-effective.

## Data Availability Statement

The raw data supporting the conclusions of this article will be made available by the authors, without undue reservation.

## Ethics Statement

The studies involving human participants were reviewed and approved by the Institutional Review Board of Zhongshan Ophthalmic Center, Sun Yat-sen University, China. The patients/participants provided their written informed consent to participate in this study.

## Author Contributions

WC, HC, HL, and WRC: concept and design and critical revision of the manuscript. WC, HC, LM, JL, HL, and WRC: data acquisition, statistical analysis, and interpretation. WC: drafting of the manuscript. WC, HC, and WRC: securing funding, technical support, supervision, and final approval of the manuscript. All authors agreed to be accountable for all aspects of the work in ensuring that questions related to the accuracy or integrity of any part of the work are appropriately investigated and resolved, read, and approved the final manuscript.

## Conflict of Interest

The authors declare that the research was conducted in the absence of any commercial or financial relationships that could be construed as a potential conflict of interest.

## Publisher’s Note

All claims expressed in this article are solely those of the authors and do not necessarily represent those of their affiliated organizations, or those of the publisher, the editors and the reviewers. Any product that may be evaluated in this article, or claim that may be made by its manufacturer, is not guaranteed or endorsed by the publisher.
